# Clinicopathological features and outcome of gastric metastases from primary lung cancer: A case report and systematic review

**DOI:** 10.3892/ol.2014.2830

**Published:** 2014-12-24

**Authors:** QINGYUAN HUANG, XIAODONG SU, AMOS ELA BELLA, KONGJIA LUO, JIETIAN JIN, SHUISHEN ZHANG, GUANGYU LUO, TIEHUA RONG, JIANHUA FU

**Affiliations:** 1 Department of Thoracic Oncology, Sun Yat-Sen University Cancer Center, State Key Laboratory of Oncology in South China, Innovation Center for Cancer Medicine, Guangzhou, Guangdong, P.R. China; 2 Guangdong Esophageal Cancer Research Institute, Sun Yat-Sen University Cancer Center, State Key Laboratory of Oncology in South China, Innovation Center for Cancer Medicine, Guangzhou, Guangdong, P.R. China; 3 Department of Pathology, Sun Yat-Sen University Cancer Center, State Key Laboratory of Oncology in South China, Innovation Center for Cancer Medicine, Guangzhou, Guangdong, P.R. China; 4 Department of Endoscopy, Sun Yat-Sen University Cancer Center, State Key Laboratory of Oncology in South China, Innovation Center for Cancer Medicine, Guangzhou, Guangdong, P.R. China

**Keywords:** clinicopathological features, primary lung cancer, gastric metastasis, outcomes, EGFR mutation

## Abstract

Primary lung cancer is the fourth most frequently diagnosed cancer, but gastric metastasis from lung cancer is extremely rare. Little is known about its clinicopathological features, prognosis and optimal treatment strategy. The present study reports a case of primary lung cancer that metastasized to the stomach and to the best of our knowledge, is the first to identify discordance in epidermal growth factor receptor (EGFR) mutation status between the primary tumor and gastric metastasis. The study also systematically searched the Medline database for similar cases to provide a literature review. Data concerning the clinicopathological features, treatment strategies and outcomes were extracted and analyzed. In total, 22 eligible cases were identified from 16 studies. The average age at presentation was 67.3 years and there was a male predominance of 90.9%. Epigastric pain (45.5%) was the most common chief complaint, followed by melena (22.7%), nausea/vomiting (13.6%) and hematemesis (9.1%). Three patients were asymptomatic. Five patients sought the initial consultation for gastrointestinal symptoms. The median time between the primary lung cancer diagnosis and the confirmation of gastric metastasis was five months. Endoscopically, gastric lesions were described as polypoid masses or volcano-like ulcers, mostly involving the gastric corpus, which were identified in 62.5% of the 16 cases in which information regarding the site of metastasis was available. Gastric metastases were reported from adenocarcinoma, squamous cell carcinoma, small cell lung cancer and pleomorphic carcinoma of the lung. The median survival following comprehensive treatment strategies was four months, and the one-year post-metastasis survival rate was 35.3%. In conclusion, although primary lung cancer metastasis to the stomach is rare, clinicians should be aware of the possibility of its occurrence. Comprehensive and personalized treatment may be beneficial to patients. EGFR tyrosine-kinase inhibitor therapy may be the treatment of choice for non-small cell lung carcinoma patients harboring an activating EGFR mutation in the metastatic lesion.

## Introduction

Based on the GLOBOCAN 2008 estimates (1), primary lung cancer accounts for 17% of newly diagnosed cancer cases and 23% of total cancer-related mortalities, and the lung is also the leading global cancer site in males. In females, lung cancer is the fourth most frequently diagnosed cancer and the second most common cause of cancer-related mortality worldwide. At the time of diagnosis, approximately half of patients have metastatic disease, with the reported post-diagnosis survival rates being 20% at one year and 1% at five years (2,3). The common metastatic sites of primary lung cancer are the liver, bones, adrenal glands and central nervous system, while gastrointestinal metastasis rarely occurs. Gastric metastasis is uncommon, and autopsy results have reported the incidence to range between 0.2 and 1.7% in different studies (4,5). Only sporadic cases of gastric metastasis have been published in past decades. At present, little is known about its clinicopathological features and prognosis, and gastric metastasis remains a challenging clinical problem.

The present study reports a case of primary lung cancer metastasizing to the stomach and provides a systematic review of the previously reported cases to study the clinicopathological features and outcome of this rare entity. Written informed consent was obtained from the patient.

## Case report

### Patient characteristics and case presentation

**Figure 1:**
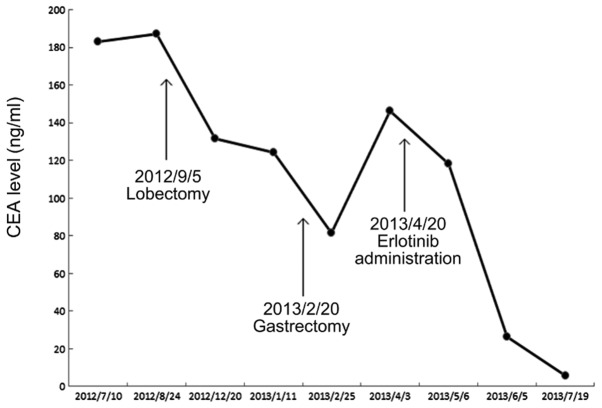
Alteration of CEA level and treatment at different time-points. The patient first presented with a raised CEA level. The CEA level gradually decreased to 5.9 ng/ml (normal value, 0–5 ng/ml) following 3 months of erlotinib treatment. CEA, carcinoembryonic antigen.

**Figure 2:**
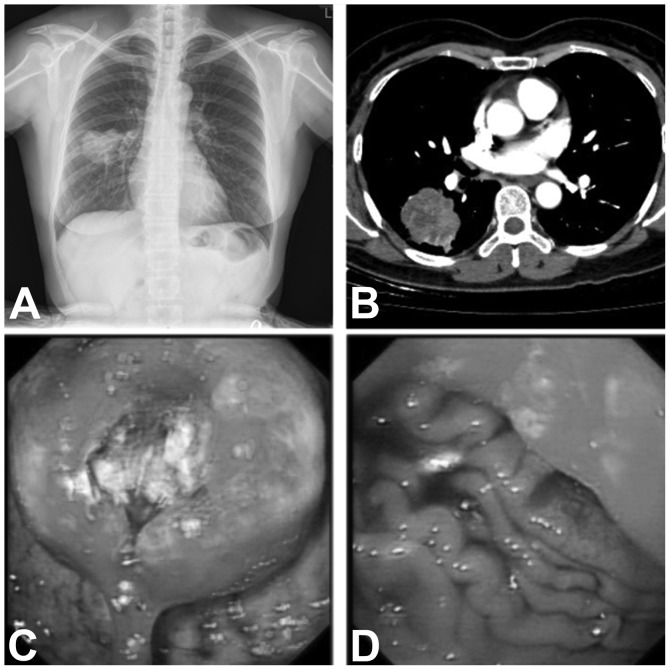
(A) Chest X-ray revealing an abnormal mass shadow in the right lower lobe. (B) Computed tomography scan of the chest demonstrating an irregular mass measuring 39×48 mm in size, with a scallop-shaped contour and focal enhancement. (C and D) Gastroscopy images revealing a mass, ~4×4 cm in size, in the fundus of the stomach, invading the cardia, with a deep ulcer in the center.

**Figure 3:**
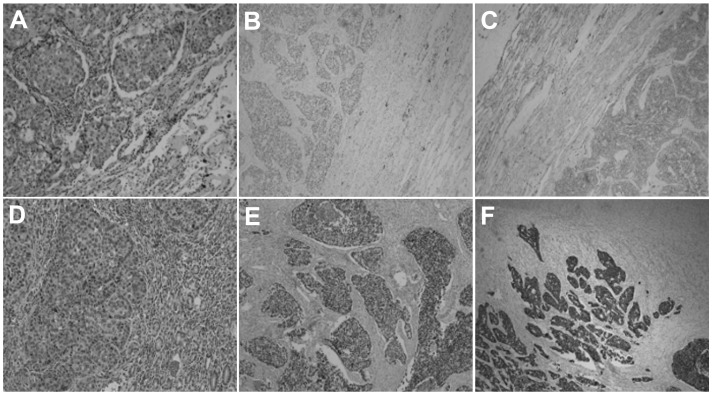
(A) Cancer tissue revealing the atypical nest and mesh shape of the cell arrangement (stain, hematoxylin and eosin; magnification, ×100). The positive immunohistochemical staining for (B) TTF-1 and (C) CK-7 indicates that the cancer originated from the lung. (D) HE staining of the gastric mass tissues (magnification, ×100). The cancer tissue exhibits similar HE morphology to lung adenocarcinoma, and there are clear boundaries between the cancer tissue and the normal gastric gland. Positive immunohistochemical staining for (E) TTF-1 and (F) CK-7 which is the same result as found in lung adenocarcinoma. TTF-1, thyroid transcription factor-1; CK, cytokeratin; HE, hematoxylin and eosin.

On a routine heath check-up in July 2012, a raised carcinoembryonic antigen (CEA) value of 183.2 ng/ml (normal value, 0–5 ng/ml; Fig. 1) was found in an asymptomatic, 61-year-old female who was a non-smoker and a non-drinker. A prominent submucosal lesion, ~0.7×0.8 cm in size, was detected in the fundus of the stomach through gastroscopic examination. An abnormal chest X-ray shadow in the right lower lobe was later detected (Fig. 2A). A computed tomography (CT) scan of the chest and abdomen revealed an irregular mass without any abdominal abnormality (Fig. 2B). A right lower lobectomy, with complete mediastinal lymph node dissection was performed. Pathological examination of the surgical specimen revealed a poorly-differentiated, stage IB (T2aN0M0), adenocarcinoma (Fig. 3A). Immunohistochemically, the tumor cells were positive for thyroid transcription factor (TTF)-1 and cytokeratin (CK)-7 (Fig. 3B and C).

Four months later, during follow-up, the patient complained of epigastric discomfort without dysphagia or melena. Laboratory examinations indicated a raised CEA level of 124.2 ng/ml. The positron emission tomography (PET)-CT scan revealed a thickening of the cardia wall, with increased fluorodeoxyglucose activity (maximum standardized uptake value, 19.4) that was consistent with malignancy. Further gastroscopy revealed a mass with a deep ulcer (Fig. 2C and D); endoscopic ultrasonography (EUS) was not performed due to fear of perforation. The biopsy confirmed the mass to be a poorly-differentiated adenocarcinoma. The patient underwent a partial gastrectomy, and the histology of the excised tissue was found to be the same as that from the biopsy (Fig. 3D). The diagnosis of gastric metastasis from primary lung cancer was confirmed immunohistochemically by positive staining for TTF-1 and CK-7 (Fig. 3E and F), and negative staining for CDX2 and Villin. The EGFR gene of the gastric metastasis harbored a 19th exon mutation, identified by the Amplified Refractory Mutation System method, which detects single base pair mutations in a background of wild-type DNA (6), while the primary lung tumor showed a wild-type EGFR sequence. Erlotinib treatment (150 mg, once a day) was commenced in April 2013. The CEA level decreased to 5.9 ng/ml in July 2013, and the patient was alive and ambulatory at the time of writing this study.

### Systematic review

A systematic review of the cases reported in the literature was conducted to examine the nature of gastric metastasis from primary lung cancer. The Medline database was searched for literature published between 1966 and 31 December, 2012. The search strategy was (‘lung cancer’ OR ‘lung neoplasms’ [MeSH Terms]) AND (‘stomach’ OR ‘gastric’ [All Fields]) AND (‘metastasis’ [All Fields]), filtering for case reports that were in English and focused on humans. All potentially eligible studies were retrieved and their references were carefully scanned to identify other eligible studies.

The systematic review included studies that fulfilled all of the following criteria: i) A focus on gastric metastasis from primary lung cancer; ii) a diagnosis verified by pathological examination; and iii) a previously unreported patient group. Criteria for excluding articles for further review were: i) Gastrointestinal metastasis without involvement of the stomach; ii) provision of insufficient clinicopathological data, such as the complaint and pathological type; and iii) autopsy studies.

**Figure 4:**
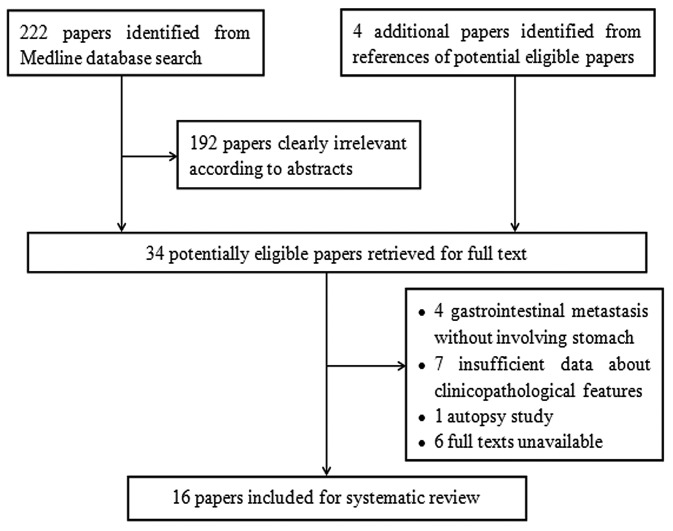
Flow diagram demonstrating the selection strategy used for the systematic review.

A total of 222 articles were retrieved by a literature search of the Medline database, using the aforementioned search strategy. As indicated in the search flow diagram (Fig. 4), a total of 16 studies were finally included. These studies were comprised of the case reports of 22 patients. Table I summarizes the patients, the tumor characteristics, the therapies implemented and the survival times recorded.

As detailed in Table I, it was determined that the average age at presentation was 67.3 years (range, 46–82 years). There were 20 males (90.9%) and two females (9.1%). Overall, 11 patients (50%) were cigarette smokers, one (4.5%) had never smoked and the smoking status of 10 patients (45.5%) was not reported.

The presenting symptoms were mainly abdominal in nature (18 patients, 81.8%), including epigastric pain, melena, hematemesis and vomiting. Some patients also presented with fever, anorexia, anemia or substernal pain. There were 10 patients whose primary cancer and gastric metastases were confirmed during the same series of work-up. For the 11 patients with gastric metastases confirmed after lung cancer, the median time span between the diagnosis of lung cancer and the detection gastric metastasis was 5 months. It is noteworthy that one patient had gastric ulcer detected 14 weeks before lung cancer was detected.

Endoscopically, two main types of gastric lesions were described: The nodular or fungating mass and the volcano-like or umbilicated ulcer with raised margins, a number of them hemorrhagic. The body of the stomach was the most common site of metastasis in 62.5% of the 16 patients in which information regarding the site of metastasis was available.

Gastric metastases were mostly from primary lung adenocarcinoma (40.9%) followed by squamous-cell carcinoma (36.4%), small cell lung cancer (13.6%) and pleomorphic carcinoma (9.1%).

**Figure 5:**
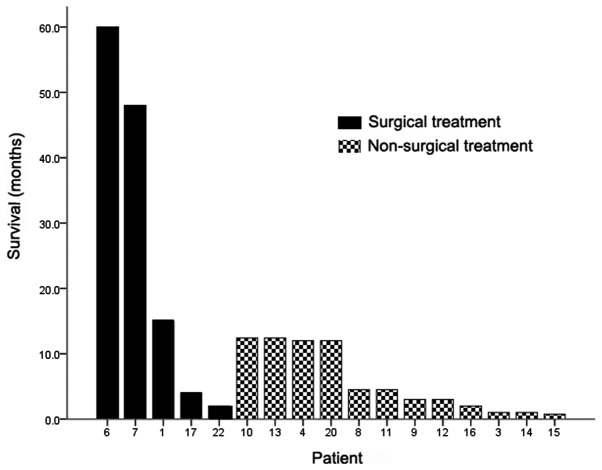
Survival of patients with gastric metastases. Patients receiving surgical treatment tended to survive longer.

Nine patients (40.9%) developed gastric metastasis as a single-site metastasis at the time of diagnosis and had no other clinically detectable metastatic lesion, whereas 10 patients (45.5%) also demonstrated other common metastatic sites of lung cancer, including the bones, brain and liver. Common treatment regimens for gastric metastases include surgery, chemotherapy or chemoradiotherapy, and supportive care. Six of the nine patients with single gastric metastasis received surgical treatment, ranging from a total, subtotal or partial gastrectomy to excision of the ulcer margin. The median survival of the 17 patients whose outcomes were available was four months, and the one-year post-metastasis survival rate was 35.3%. Three of the five patients who were treated surgically for solitary gastric metastasis survived for more than one year following confirmation of the metastasis (Fig. 5).

## Discussion

The identification of gastric metastasis from lung cancer in a female patient who had never smoked was rare and incidental in the present study. There is a high rate of discrepancy between the clinical and autopsy diagnoses of gastric metastasis from primary lung cancer, as the majority of the cases are detected during autopsy (20). Therefore, it may be estimated that a high number of gastric metastases remain asymptomatic and clinically undetectable.

Among the symptomatic cases in the present literature review, epigastric pain was the most common chief complaint, followed by upper gastrointestinal bleeding (melena and hematemesis), nausea/vomiting and general weakness or fatigue. Gastric perforation due to metastasis was rare. These symptoms are not specific and are usually regarded as side-effects of chemotherapy or as symptoms of the involvement of the central nervous system. This fact makes gastric metastasis occasionally difficult to confirm (14). With the rapid development of comprehensive treatment and supportive care for lung cancer, and the subsequent survival benefit, an increasing number of rare types of metastatic disease from primary lung cancer are likely to be encountered (2), and clinicians therefore ought to be aware of the possibility of their occurrence. Patients with unspecific gastrointestinal symptoms following chemotherapy or during the follow-up should be carefully monitored, and gastroscopy should be performed when necessary.

Detection of a gastric abnormality is usually incidental during the follow-up or the staging procedures of primary lung cancer, and occasionally, detection could be even earlier than that for lung cancer. Taking the present case and five other cases (15–19) as examples, the primary lung lesions were recorded as being found on chest X-ray following referral to hospital for a gastric abnormality. There is a risk of misdiagnosis associated with gastric metastases, since they can manifest prior to the identification of the primary malignancy and may be misdiagnosed as primary gastric cancer, so that consequently, the true primary malignancy is not recognized (19).

Numerous gastric tumors consist of mucosal and submucosal elements. Metastatic lesions in certain patients can invade the submucosa or muscular layer, rather than the mucosa (6). Besides the clinical manifestations, the morphology of gastric metastasis observed on gastroscopy could mimic that of other gastric tumors. There are no typical appearances that define metastatic disease. Therefore, EUS should be considered to be a powerful diagnostic tool in gastric lesions, as it can determine the depth of invasion of the gastric wall (22).

A nodular or fungating mass and a volcano-like or umbilicated ulcer are the two types of endoscopic appearance of a gastric metastatic malignancy. The lesion in the current case presented as a submucosal nodule on the first gastroscopy, which evolved to form a deep ulcer six months later upon reexamination by gastroscopy. This phenomenon suggests that gastric metastases may exhibit different appearances in connection with the stage of the disease. In early stages, they could appear as nodules. Following considerable growth, the metastases invade the mucosa and ulceration develops. These lesions have previously been reported to be usually located on the fundus (25), while results from the present study and a study by Wu *et al* (20) have suggested that the body of the stomach is the most common site of metastasis.

In the present literature review, when gastric metastasis was diagnosed, the metastasis was associated with other organs in more than half of the lung cancer patients. The prognosis of patients with gastric metastasis following complete non-small cell lung carcinoma (NSCLC) resection is generally poor and only approximately one in three patients survive longer than one year. The one-year post-metastasis survival rate in the present literature review was close to that of patients with extrathoracic recurrence following complete NSCLC resection, with a reported one-year post recurrence survival rate of 26% (24).

Comprehensive and personalized treatment should be the treatment strategy for gastric metastases from primary lung cancer. Systematic chemotherapy with or without radiotherapy would be the first option for selected patients, and molecular targeted therapy may also be a reasonable choice if the patient was found to possess an EGFR mutation or to be EML4-ALK-positive. Patients with a poor performance status should be provided with supportive treatment to improve the quality of life.

Generally, a distant metastatic lesion that has originated from lung cancer is a contraindication for surgical therapy. However, resection of a solitary metastatic lesion in the brain or adrenal gland is becoming the standard of care that has exhibited a survival benefit. In NSCLC patients with a single metastasis other than metastasis in the brain or adrenal gland, Salah *et al* (25) found that metastasectomy significantly prolonged the five-year overall survival rate. Additionally, a previous study reported long-term survival following resection of solitary gastric metastases. Aokage *et al* (21) observed two patients with solitary gastric metastases from pulmonary pleomorphic carcinoma who survived for four years and five years after surgery, respectively. According to the present review data, patients receiving surgical treatment for solitary gastric metastases tended to survive longer than others. Since literature data on the surgical treatment of single metastasis is scant, and more cases are necessary to evaluate the effectiveness of the surgical treatment for gastric metastasis from lung cancer. In addition, surgical intervention is usually indicated when gastric metastasis leads to continuous bleeding or perforation.

Locally advanced (stage III) or metastatic NSCLC patients with activating mutations in the EGFR gene have exhibited a dramatic response to EGFR tyrosine kinase inhibitors (EGFR-TKI), such as gefitinib and erlotinib, since these activating mutations, including exon 19 deletions and the L858R point mutation in exon 21, are recognized as markers of EGFR-TKI therapy sensitivity in NSCLC (26). Activating EGFR mutations predominate in never-smokers, females and tumors with adenocarcinoma histology (27). For the present patient, an EGFR gene mutation in exon 19 was detected in the gastric metastasis, and therefore, erlotinib was administered. The primary lung adenocarcinoma, however, harbored a wild-type EGFR sequence. This mismatch is not novel; previous studies have revealed discordances in EGFR status between the primary tumor and the corresponding metastases in approximately one-third of cases (28–30). Han *et al* also proved that a significant portion of lung adenocarcinoma exhibits discordances in EGFR mutation between primary tumors and the corresponding metastases (31). Gow *et al* (28) indicated that, in the majority of discordant cases, the primary tumor possessed wild-type EGFR while the corresponding metastasis possessed the EGFR mutation. This suggests that the molecular properties of EGFR are not stable and are likely to change during the process of lung cancer metastasis (29,32). However, the prognostic role of EGFR in metastatic gastric cancer has yet to be established. To the best of our knowledge, identification of the EGFR gene mutation in the gastric metastasis of a primary lung cancer patient with wild-type EGFR has not been reported to date. The gradually decreasing CEA level following erlotinib administration indicates that erlotinib is beneficial for this type of patient.

In selecting lung cancer patients with solitary gastric metastasis for specific targeted therapies by EGFR analysis, special attention should be given to the metastatic lesions rather than their corresponding primary tumors. EGFR-TKI therapy may be a reasonable treatment for NSCLC patients harboring an activating EGFR mutation in the metastatic lesion.

Primary lung cancer metastasizing to the stomach is rare, however, clinicians should be aware of the possibility of its occurrence. Comprehensive and personalized treatment may be beneficial to such affected patients. EGFR TKI therapy may be the treatment of choice for NSCLC patients harboring an activating EGFR mutation in the metastatic lesion.

## Figures and Tables

**Table I:** Clinicopathological features and outcome of the 22 cases.

Patient	First author, year (ref.)	Gender	Age, years	Smoking	Main Complaint	Time span, months	Endoscopic features	Gastric metastatic site	Histology	Primary lung site	Treatment	Survival, months
1	Sileri, 2012 (6)	Male	68	Y	Epigastric pain with nausea and anorexia	48.0	Neoplasm, originating from muscular layer	Pylorus	AC	RUL	Subtotal gastrectomy	>15
2	Lee, 2010 (7)	Male	77	NR	None	0.0	Ulcer with raised margin	Pylorus	AC	RUL	Lobectomy + subtotal gastrectomy	NR
3	Ozdilekcan, 2010 (8)	Male	46	Y	Disphagia and epigastric pain	0.5	Giant ulcer	Body	SCC	RUL	CRT	1.00
4	Okazaki, 2010 (15)	Male	68	Y	Epigastric pain	0.0	Erosive tumor	Body	AC	RLL	CT	12.00
5	Kanthan *et al*, 2009 (19)	Male	75	N	Epigastric and right upper quadrant pain	0.0	Polyps	NR	AC	Right	NR	NR
6	Aokage *et al*, 2008 (21)	Male	69	NR	General fatigue and anemia	5.0	Hemorrhagic tumor	Body	Pleomorphic carcinoma	RUL	Distal gastrectomy	60.00
7		Male	62	NR	None	0.0	NR	Fundus	Pleomorphic	LUL	Partial gastrectomy and splenectomy	48.00
8	Wu *et al* (20)	Male	73	NR	Melena	108.0	NR	Cardia	SCC	NR	Conservation	1.00
9		Male	82	NR	Melena	5.0	NR	Pylorus	AC	NR	Conservation	1.00
10		Male	70	NR	Epigastric pain	5.0	NR	Body	AC	NR	Conservation	10.0
11	Yang *et al*, 2006 (2)	Male	71	Y	Melena	0.0	NR	NR	SCC	LUL		4.50
12		Male	65	Y	Melena	0.0	NR	NR	SCC	RML		3.00
13		Male	62	Y	Melena	1.7	NR	NR	AC	RUL		12.40
14	Casella *et al*, 2006 (16)	Male	63	Y	Fever and epigastric pain	0.0	A raised area depressed on the tip	Pylorus	Small cell cancer	LUL	Supportive care	1.00
15	Altintas, 2006 (9)	Male	55	NR and melena	Hematemesis	11.0 lesions	Two volcano-like	Body	AC	NR	CRT	0.75
16	Alpar, 2006 (10)	Male	66	Y	Epigastric pain and vomiting	3.0	Erosive and atrophic pangastritis	NR	SCC	NR	None	2.00
17	Hamatake, 2001 (11)	Male	65	Y	Acute hematemesis	3.0	Bleeding gastric ulcer	Body	SCC	LLL	Total gastrectomy	4.00
18	Kim *et al*, 1993 (17)	Male	66	NR	Epigastric pain and weakness	0.0	Multiple submucosal lesion with umbilications	Body and fundus	Small cell cancer	LUL	NR	NR
19		Male	68	Y	None	0.0	Fungating mass	Body	SCC	LUL	NR	NR
20	Fukuda *et al*, 1992 (12)	Female	79	NR	Epigastric pain	0.0	Submucosal tumor with central ulceration	Fundus	AC	RLL	NR	12.00
21	Maeda *et al*, 1992 (14)	Female	60	NR	Nausea and vomiting	3.0	Multiple submucosal tumors	NR	Small cell cancer	RLL	Conservation	NR
22	Fletcher, 1980 (18)	Male	70	Y	Epigastric and substernal pain	3.5 prior	Raised mucosa with ulceration	Body	SCC	LLL	Ulcer excision and truncal vagotomy	2.00

Time span, the time elapsed between the diagnoses of primary lung cancer and gastric metastasis; AC, adenocarcinoma; SCC, squamous cell carcinoma; CRT, chemoradiotherapy; LLL, left lower lobe; LUL, left upper lobe; RUL, right upper lobe; RML, right middle lobe; RLL, right lower lobe; NR, not reported; Y, yes; N, no.
